# Supplementary material for: Imaging moiré deformation and dynamics in twisted bilayer graphene
Source: Nat Commun. 2022 Jan 10;13:70. doi: 10.1038/s41467-021-27646-1 (PMC8748992; doi:10.1038/s41467-021-27646-1)
Supplement: Supplementary file 1 — Supplementary Information [file 41467_2021_27646_MOESM1_ESM.pdf]

Supplementary information to "Imaging moiré deformation and dynamics in twisted bilayer graphene"

Tobias A. de Jong[1,*], Tjerk Benschop[1], Xingchen Chen[1], E.E. Krasovskii[2,3,4], Michiel J.A. de Dood[1], Rudolf M. Tromp[5,1], Milan P. Allan[1], Sense Jan van der Molen[1,*]

[1]Leiden Institute of Physics, Leiden University, P.O. Box 9504, 2300 RA Leiden, The Netherlands
[2]Departamento de Polímeros y Materiales Avanzados: Física, Química y Tecnología, Universidad del Pais Vasco UPV/EHU, 20080 San Sebastián/Donostia, Spain
[3]IKERBASQUE, Basque Foundation for Science, E-48013 Bilbao, Spain
[4]Donostia International Physics Center (DIPC), E-20018 San Sebastián, Spain
[5]IBM T.J.Watson Research Center, 1101 Kitchawan Road, P.O. Box 218, Yorktown Heights, New York, New York 10598, USA December 2, 2021

Supplementary figures

Additional LEEM images/crops

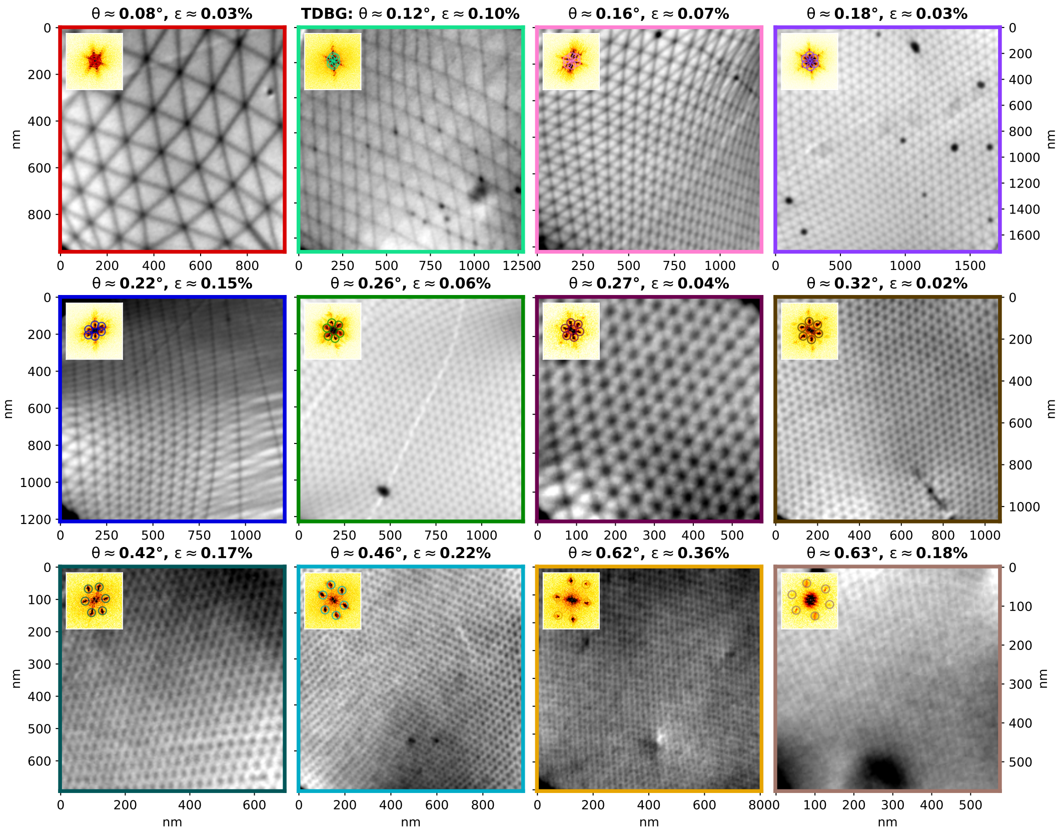

Supplementary Figure 1: Supplementary moiré LEEM crops. *A wider range of images found in the sample from the main text as used to determine the histograms of twist angles and strain in Figure 2 of the main text. Insets show FFT's with the detected moiré peaks.*

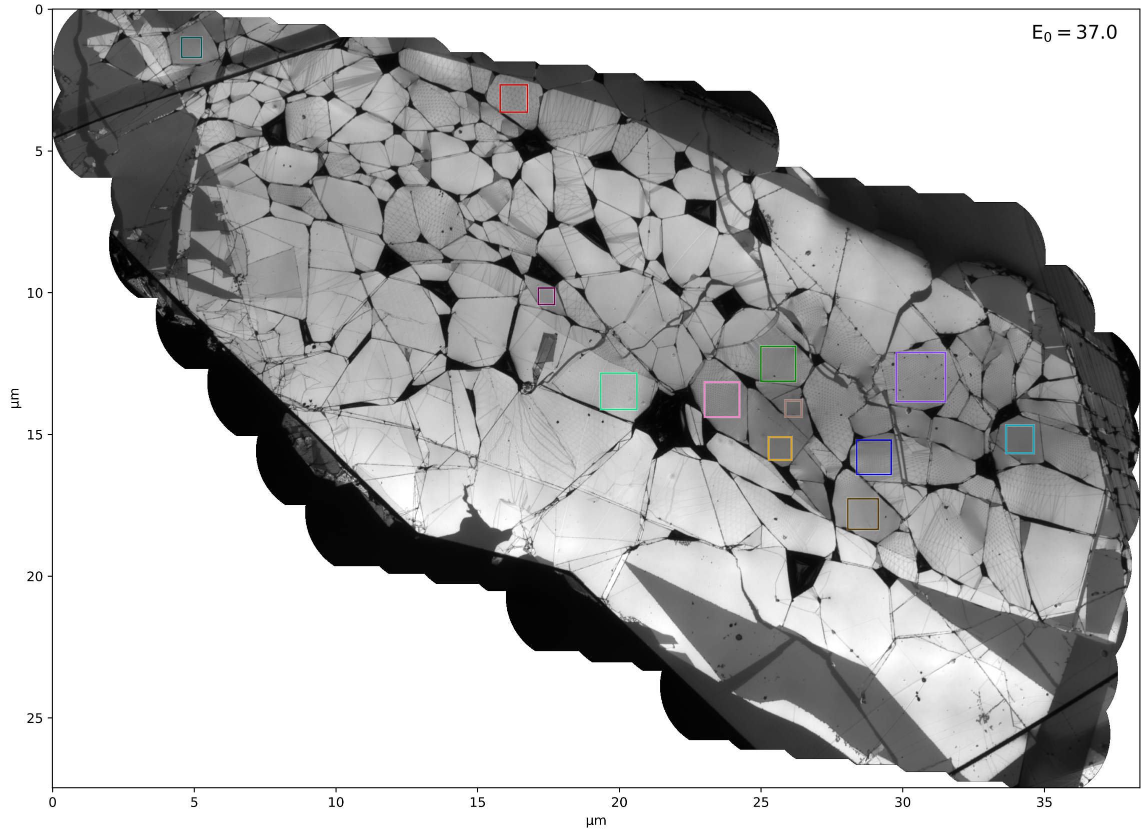

Supplementary Figure 2: Crop locations. *Locations of the crops in Figure 1 indicated in the full overview (data is the same as Figure 1**e** of the main text).*

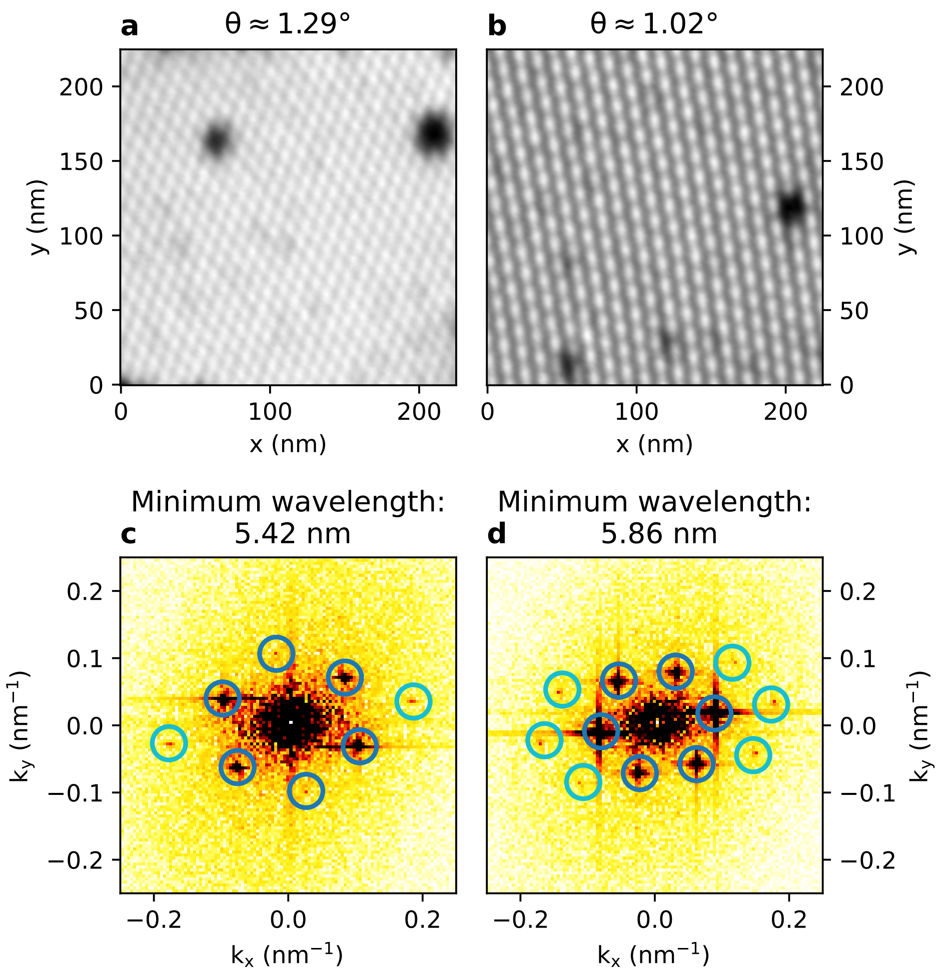

Supplementary Figure 3: LEEM moiré resolution. ***a,b,*** *Images of 2-on-2 layer twisted graphene at* $E_0 = 17.0\,\mathrm{eV}$*, with twist angles of respectively* $\theta \approx 1.29°$ *and* $\theta \approx 1.02°$ ***c,d,*** *FFTs of a,b with Bragg peaks corresponding to the moiré pattern indicated in blue. Higher order moiré peaks are also visible (indicated in cyan), corresponding to minimum detectable wavelengths of less than* $6\,\mathrm{nm}$.

Supplementary figures on dynamics

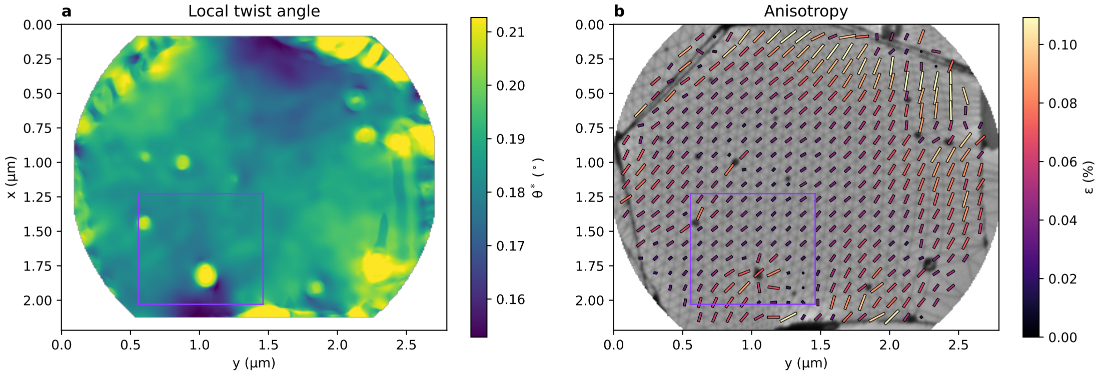

Supplementary Figure 4: Local properties at the movie location. ***a,*** *Local twist angle as extracted with GPA of the area imaged in Supplementary Video 1.* ***b,*** *Local strain magnitude and direction as extracted with GPA of the same area. Purple rectangles indicate the area depicted in Figure 4 of the main text.*

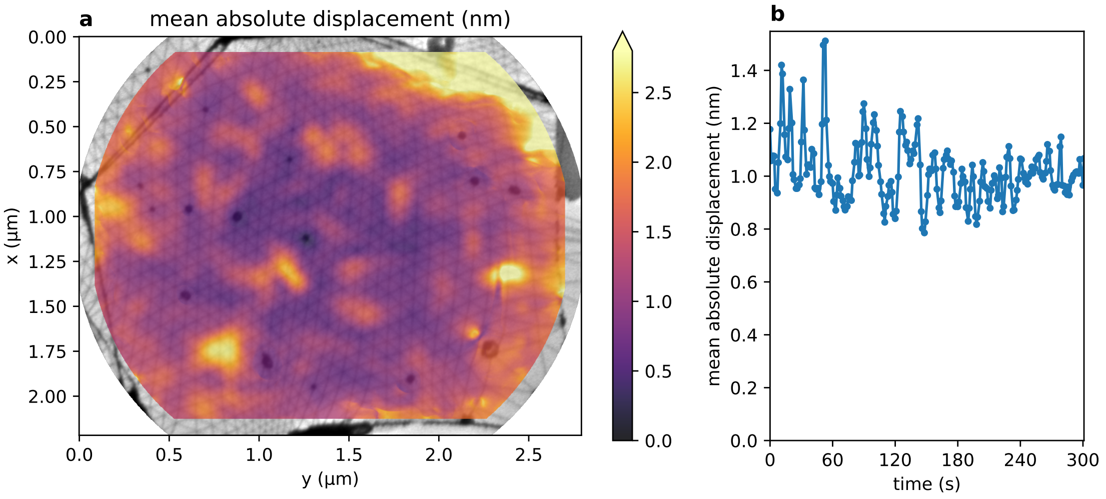

Supplementary Figure 5: Moiré dynamics statistics. ***a,*** *(Temporal) Mean absolute displacement from mean position during Supplementary Video 1.* ***b,*** *Spatial mean absolute displacement from mean position during Supplementary Video 1 as a function of time for a center area.*

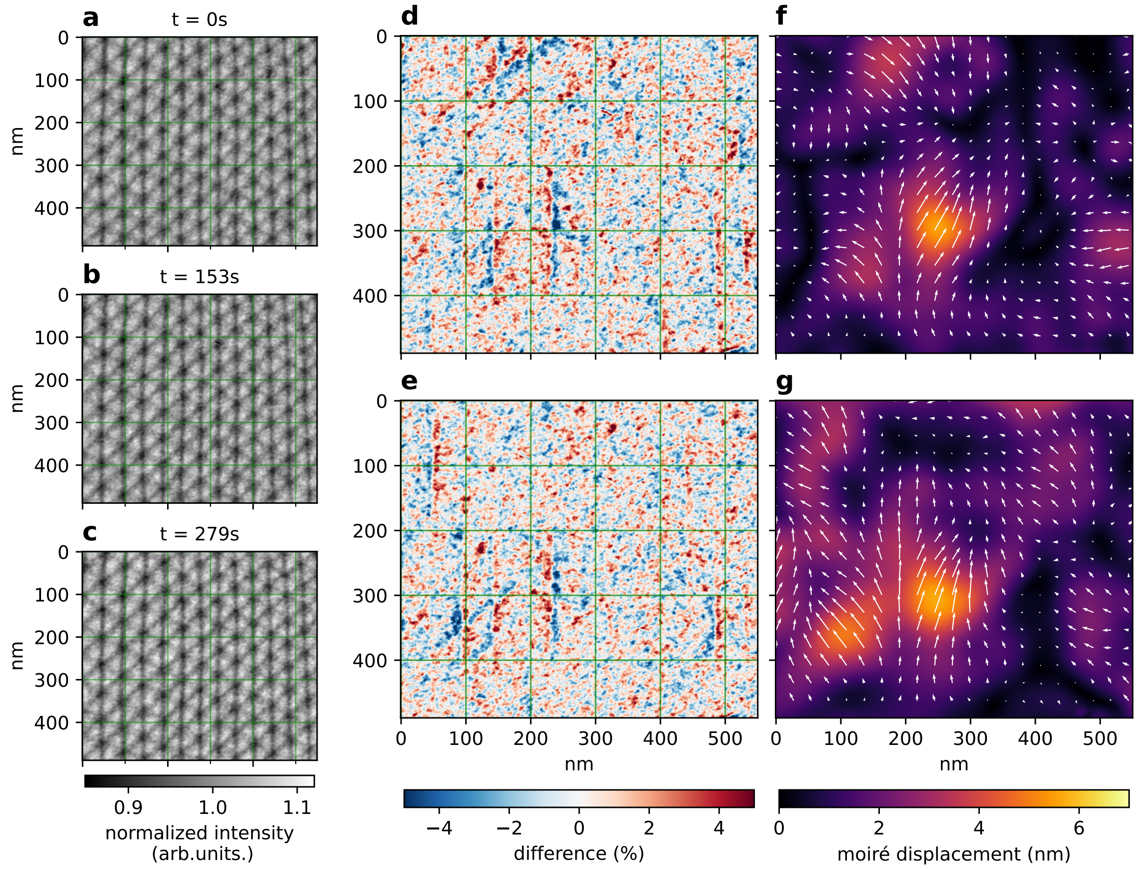

Supplementary Figure 6: Dynamics of an additional area. **a-c,** *Three images of the same area (in the same domain as Supplementary Figure 1***f***), taken minutes apart at a constant temperature of 500°C. Here, $\theta^* \approx 0.26$ and $\epsilon \approx 0.08\%$ (local values as extracted by GPA shown in Supplementary Figure 7).* **d,e,** *Difference of respectively* **b** *and* **c** *with* **a***, i.e. $t = 0\,$s, highlighting the shift of the domain boundaries.* **f,g,** *GPA extracted displacement of respectively* **b,** *and* **c,** *with respect to $t = 0\,$s, with the arrows indicating the direction and amplitude magnified 8 times for visibility.*

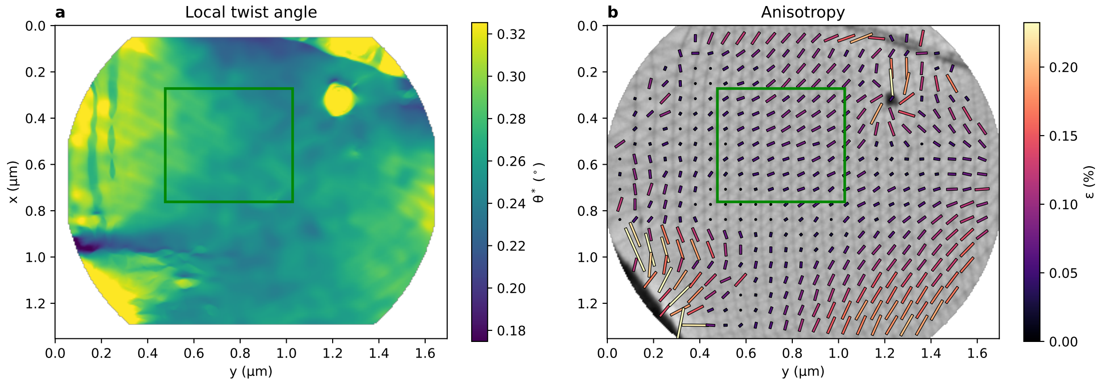

Supplementary Figure 7: Local properties. **a,** *Local twist angle as extracted with GPA of the area shown in Supplementary Figure 6.* **b,** *Local strain magnitude and direction as extracted with GPA of the same area. Green rectangles indicate the area depicted in Supplementary Figure 6.*

Supplementary figures on dislocations

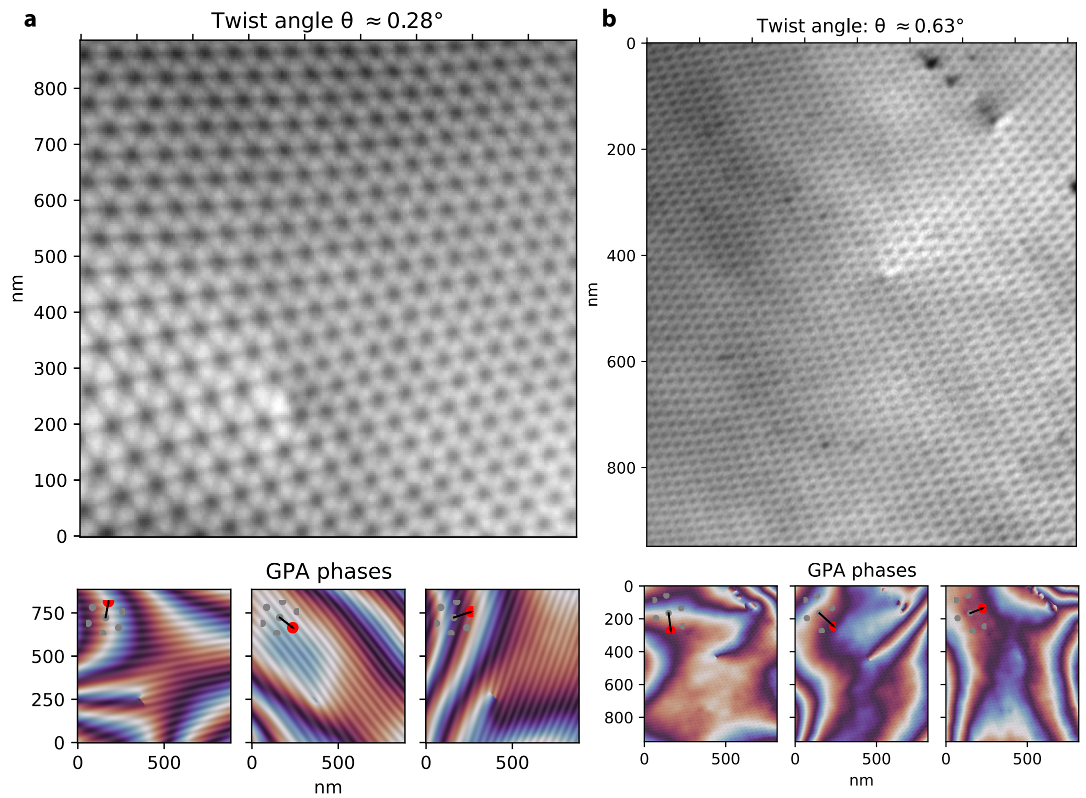

Supplementary Figure 8: Isolated edge dislocations. **a,** *Additional edge dislocation found on the sample at a lower twist angle.* **b,** *Larger area around edge dislocation in the main text from the main text. In both case GPA phases are also displayed.*

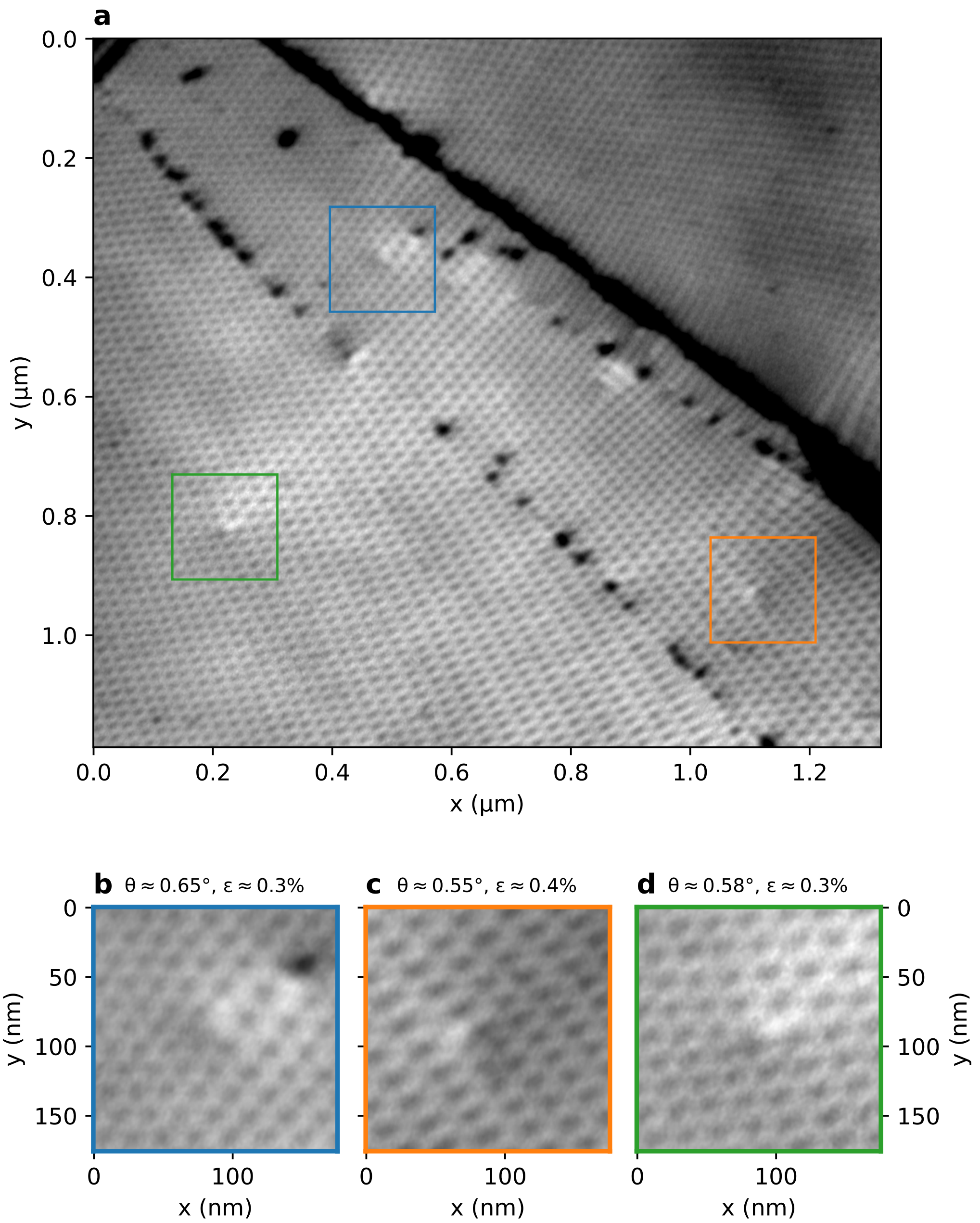

Supplementary Figure 9: Additional edge dislocations. *More dislocations in the vicinity of the dislocation shown in the main text.* ***d,*** *corresponds to the dislocation in the main text.* θ *as extracted from the shown area here is a bit lower as unit cell area tends to be a bit larger near the dislocation.*

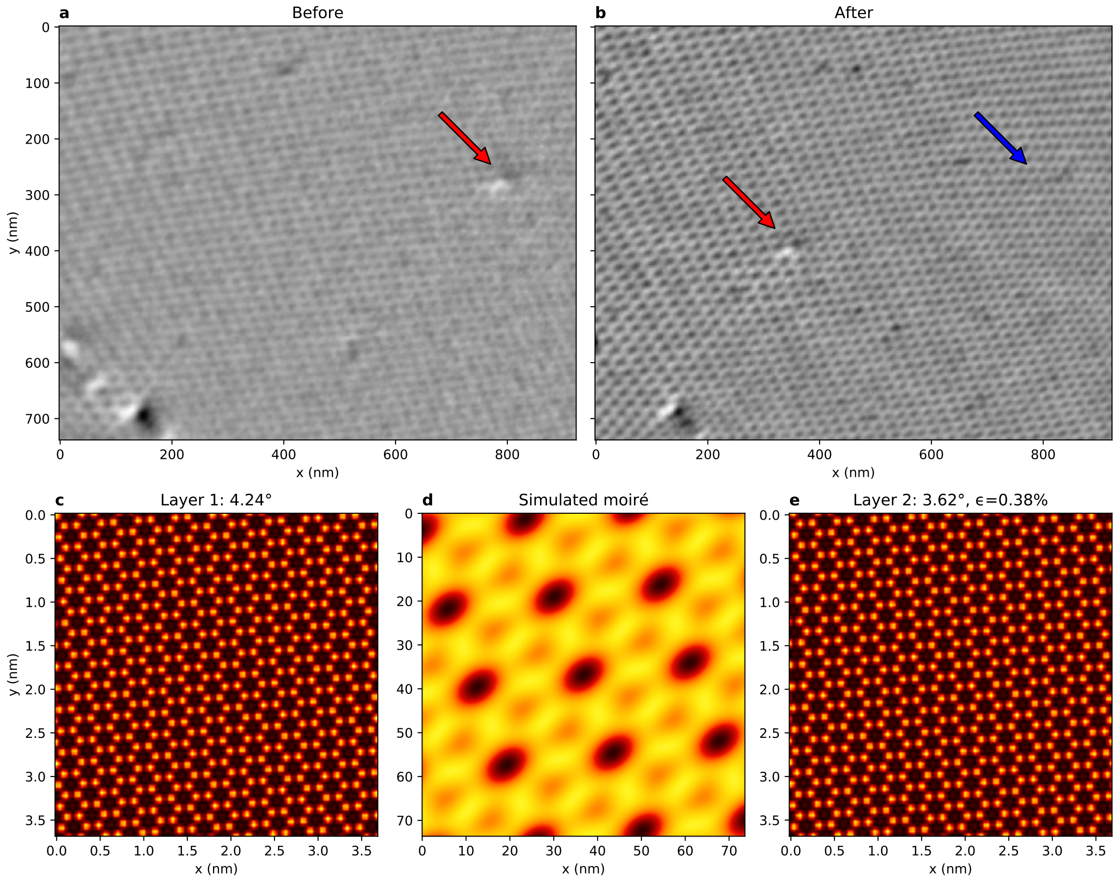

Supplementary Figure 10: Movement of dislocation. ***a,*** *Dislocation in its original location, indicated by red arrow.* ***b,*** *Image of the same area as in* ***a,****, but imaged two days later. The dislocation has moved, as indicated by the red arrow. The former location is indicated with a blue arrow.* ***c-e,*** *Rendering of the individual atomic lattices and the resulting moiré lattice from the extracted lattice parameters, showing the atomic lattice directions.*

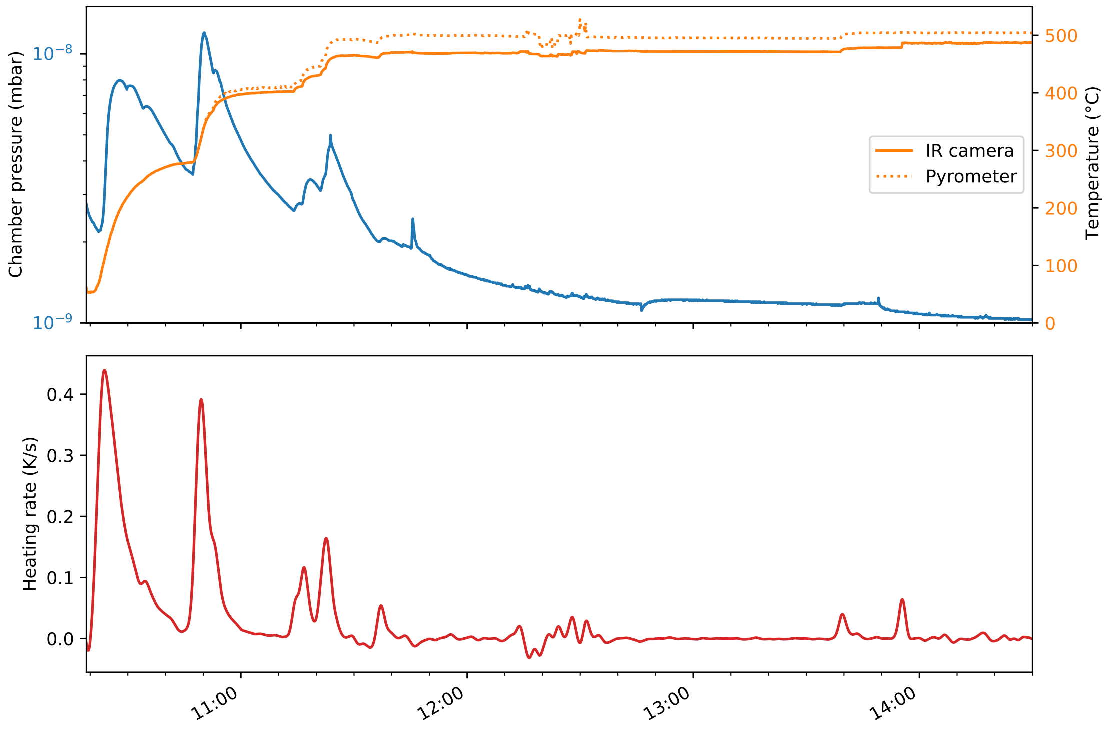

Supplementary Figure 11: Sample heating. *Sample temperature, heating rate and chamber pressure as measured by pyrometer and IR camera during initial heating.*

Supplementary Note 1: AFM data

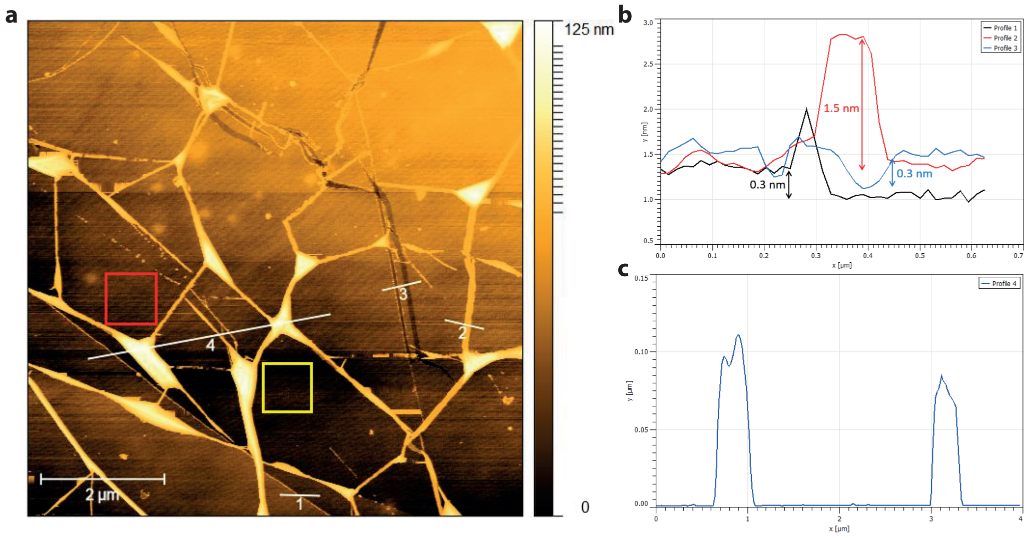

Supplementary Figure 12: AFM large area characterization. ***a,*** *Atomic Force Microscopy overview of sample area. Locations of line profiles and detailed topographies in Figure 13 are indicated.* ***b,c,*** *Line cuts along the cuts indicated in* ***a****.*

To further characterize the surface properties of the sample, an AFM (JPK, NanoWizard 3) measurement was performed in AC tapping mode following the LEEM measurements. Predominantly, the results show a very flat and clean graphene surface between folds, indicating annealing

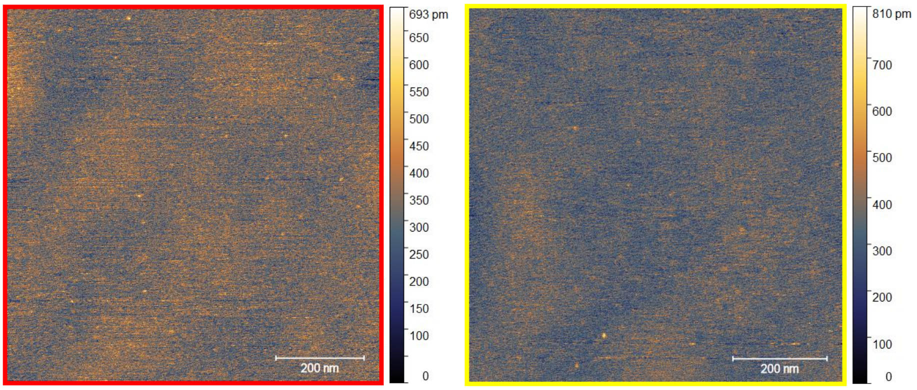

Supplementary Figure 13: AFM at edge dislocations. ***a,*** *Atomic Force Microscopy of the dislocation area in Figure 8****a****. Area is indicated in red in Figure 12****a****.* ***b,*** *Atomic Force Microscopy of the dislocation area in Figure 8****b****. Area is indicated in yellow in Figure 12****a****.*

at 500 °C in UHV had successfully removed the polymer residue left on the surface.

In profile 1, the terrace height sees a difference of 0.3 nm, demonstrating the graphene layer count goes down by one at this location. This corresponds to the layer counts extracted from the LEEM spectra.

Profile 2 to 4 shows 3 different kinds of defects in the bilayer graphene region. The ridge at location 2 seems to be a neat folding of both the bilayer graphene flake (1.5 nm in height, four layers of graphene), whereas profile 4 shows wrinkles that are up to 120 nm tall. This is also reflected by the distinct patterns in the LEEM bright field overview image, respectively. While the wrinkles merely appear black, the ridge resembles more like a unique layer count domain. Profile 3 shows two tears within the one layer of graphene, corresponding nicely to the defect region observed in LEEM where monolayer graphene shines through.

The zoomed-in small scale measurements marked by the red and yellow box shows the topography on top of two dislocations observed in LEEM. As shown in Figure 13, no distinctive feature was observed at either dislocation. The topography, however, shows an exceptionally flat surface with a height variation (peak-to-peak) of less than 1 nm.

Supplementary Note 2: On Adaptive GPA

Regular GPA is limited in the wave vector deviations (with respect to the reference wave vector) it can measure, due to the limitations in spectral leakage. This is no problem when applied to atomic lattices, as the expected deviations are very small there. However, due to the moiré magnification of small lattice distortions, it does become a limiting factor when applying GPA to small twist angle moiré lattices.

To overcome this limitation, we extended the GPA algorithm to use adaptive reference wave vectors, based on the combination of two ideas and related to earlier work in laser fringe analysis [1]: First, a GPA phase calculated with respect to one reference vector can always be converted to the GPA phase with respect to another reference vector by adding a phase corresponding to the phase difference between the reference vectors. Second, a larger lock-in amplitude corresponds to a better fit between the reference vector and the data.

The adaptive GPA algorithm therefore works as follows: The spatial lock-in signal is calculated for a grid of wave vectors around a base reference vector, converting the GPA phase to reference the base reference vector every time. For each pixel, the spatial lock-in signal with the highest amplitude is selected as the final signal.

It was realized that to deduce the deformation properties, reconstruction to a globally consistent phase (requiring 2D phase unwrapping), as reported previously [2], is not strictly needed, making it possible to circumvent the problems associated with 2D phase unwrapping. Instead, the gradient of each GPA phase was calculated, requiring only local 1D phase unwrapping (i.e. assuming the derivative of the phase in both the x and y direction will never be more than π per pixel, an

assumption in practice always met). Subsequently, these three GPA gradients are converted to the displacement gradient tensor (in real space coordinates), estimating the transformation via weighted least squares, using the local spatial lock-in amplitudes as weights.

As an added benefit, this entire procedure is local, i.e. not depending on pixels beyond nearest neighbors in any way except for the initial Gaussian convolution in determining the GPA. This reduces the effect of artefacts in the image to a minimum local area around each artefact (where for in the 2D phase unwrapping they have a global influence on the phases).

However, when the gradient is computed based on phase values stemming from two different GPA reference vectors, i.e. at the edge of their valid/optimal regions, artefacts appear due to their relatively large absolute error. To prevent this, the local gradient of the phase with the highest lock-in magnitude is stored alongside the lock-in signal itself in the GPA algorithm. This way, the gradient is calculated based on a single reference phase, propagating only the much smaller relative/derivative error between the two signals instead of the absolute error.

As mentioned in the main text, even adaptive GPA has its limits. In particular, too large deviations from the base reference vector can not be resolved correctly, causing an erroneous, lower, extracted deviation, as is visible in the lower right of Figure 2e in the main text). As the deformation becomes too large, e.g. towards the folds in the TBG, the highest lock-in amplitude will occur at a different moiré peak or at the near-zero components of the fourier transform, causing an incorrect value to be extracted.

Supplementary Note 3: On the decomposition of the displacement field.

Kerelsky et al. [3] use the following idea to extract twist angle θ_T, strain magnitude ϵ and strain direction θ_s from reciprocal moiré lattice vectors K_{is}: These difference vectors of the constituting atomic lattices are written in terms of a rotated and a strained lattice vector each:

$$K_{is} = k_{ir} - k_{is} = R(\theta_T)k_i - S(\theta_s, \epsilon)k_i$$

where k_i are the original lattice vectors. Kerelsky et al. assume k_0 to be along the x-axis, and get around this by taking amplitudes, discarding any global rotation. Here, we do however introduce that global rotation, by a multiplication with $R(\xi)$:

$$K_{is} = (R(\theta_T) - S(\theta_s, \epsilon))\, R(\xi)k_i$$

Eihter of these expressions can, and indeed by Kerelsky et al. is, numerically fitted to the found amplitudes or k-vectors for each triangle. However, from GPA analysis we most naturally obtain a Jacobian transformation J_{ac} of the moiré k-vectors with respect to some specific set of reference vectors with predefined strain and rotations:

$$K_{is} = (J + I)K_{i0} = J_{ac}K_{i0} = J_{ac}\,(R(\theta_{T0}) - S(\theta_{s0}, \epsilon_0))\, R(\xi_0)k_i := J_{ac}A_0R(\xi_0)k_i$$

Note that we can force $\epsilon_0 = 0 \rightarrow S(\theta_{s0}, \epsilon_0) = I$.
This simplifies to:

$$J_{ac}A_0R(\xi_0)k_i = (R(\theta_T) - S(\theta_s, \epsilon))\, R(\xi)k_i$$

The linear transformation is uniquely described by its effect on two points in k-space, so their matrix representations should be equal:

$$\begin{aligned} J_{ac}A_0R(\xi_0) &= (R(\theta_T) - S(\theta_s, \epsilon))\, R(\xi) \\ J_{ac}A_0 &= (R(\theta_T) - S(\theta_s, \epsilon))\, R(\xi - \xi_0) \end{aligned}$$

The left hand side is a known quantity at each position, the right hand side remains to be numerically fitted or extracted. This is implemented in `pyGPA` using `scipy.optimize` and `numba` to just-in-time compile the fitting code [4, 5].

Alternatively, we could formulate a symmetric expression with two strains, but without allowing for further joint rotation of the lattices:

$$K_{i0} = (R(\theta_{T0}/2) - R(-\theta_{T0}/2))\, R(\xi_0)k_i := B_0 R(\xi_0)k_i$$

$$J_{ac}B_0R(\xi_0)k_i = (S(\theta_b, \epsilon_b)R(\theta_T/2) - S(\theta_a, \epsilon_a)R(-\theta_T/2))\, R(\xi_0)k_i$$
$$J_{ac}B_0 = (S(\theta_b, \epsilon_b)R(\theta_T/2) - S(\theta_a, \epsilon_a)R(-\theta_T/2))$$

Supplementary Note 4: LEEM stitching

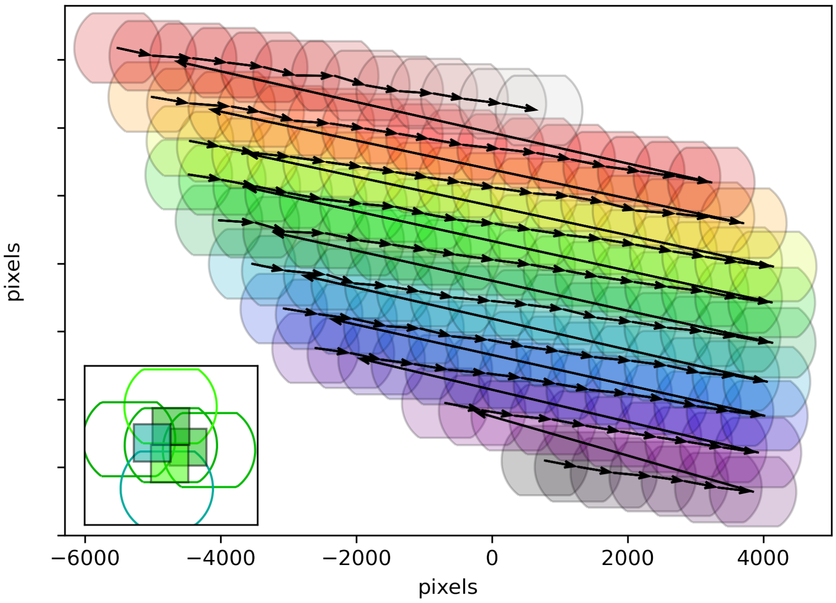

Supplementary Figure 14: Stitching schematic. *Illustration of the sample stage scanning for stitched overview images. Black arrows indicate the direction of stage movement.* ***inset,*** *Illustration of the square overlapping regions of neighboring images used to determine relative positions.*

To achieve stitching of images without inducing any additional deformation, a custom stitching algorithm tailored towards such LEEM data, was developed, working as follows:

To compensate sample stage inaccuracy, nearest neighbor (by sample stage coordinates) images are compared, finding their relative positions by cross-correlation. Using an iterative procedure, calculating cross correlations of overlapping areas at each step, the absolute positions of all images are found. Images are then combined in a weighted fashion, with the weight sloping to zero at the edges of each image, to smooth out any mismatch due to residual image warping. The full stitching algorithm is implemented in `Python`, available as a `Jupyter Notebook`[6].

It is designed for use with ESCHER LEEM images. For those images, their positions are known approximately in terms of *stage coordinates*, i.e. the positions as reported by the sensors in the sample stage. It should however generalize to any set of overlapping images where relative positions of the images are known in some coordinate system which can approximately be transformed to coordinates in terms of pixels by an affine transformation (rotation, translation, mirroring).

The algorithm consists of the following steps:

1. Using the stage coordinates for each image, obtain a nearest neighbour graph with the nearest `n_neighbors` neighbouring images for each image.

2. Obtain an initial guess for the transformation matrix between stage coordinates and pixel coordinates, by one of the following options:

   1. Copying a known transformation matrix from an earlier run of a comparable dataset.
   2. Manually overlaying some nearest neighbor images from the center of the dataset, either refining the estimate, or making a new estimate for an unknown dataset

3. Calculate an initial estimate of the pixel coordinates of the images by applying the corresponding transformation to the stage coordinates

4. Apply a gaussian filter with width `sigma` to the original dataset and apply a magnitude sobel filter. Optionally scale down the images by an integer factor `z` in both directions to be able to reduce `fftsize` by the same factor, without reducing the sample area compared.

5. Iterate the following steps until the calculated image positions have converged to within `sigma`:

   1. Obtain a nearest neighbour graph with per image the nearest `n_neighbors` neighbouring images from the current estimate of the pixel coordinates and calculate the difference vectors between each pair of nearest neighbours.
   2. For each pair of neighboring images:
      i. Calculate the cross-correlation between areas estimated to be in the center of the overlap of size `fftsize*fftsize` of the filtered data. If the estimated area is outside the valid area of the image defined by `mask/radius`, take an area as close to the intended area but still within the valid area as possible.
      ii. Find the location of the maximum in the cross-correlation. This corresponds to the correction to the estimate of the difference vector between the corresponding image position pair.
      iii. Calculate the weight of the match by dividing the maximum in the cross-correlation by the square root of the maximum of the auto-correlations.
   3. Compute a new estimate of the difference vectors by adding the found corrections. Reconvert to a new estimate of pixel coordinates by minimizing the squared error in the system of equations for the positions, weighing by modified weights, either:
      i. $w_{mod} = w - w_{min}$ for $w > w_{min}$, $w = 0$ else, with w_{min} the maximum lower bound such that the graph of nearest neighbours with non-zero weights is still connected
      ii. Only use the 'maximum spanning tree' of weights, i.e. minus the minimum spanning tree of minus the weights, such that only the n best matches are used.

6. (Optional) Refine the estimate of the transformation matrix, using all estimated difference vectors with a weight better than w_{minest} and restart from step 3.

7. Repeat step 4. and 5. until `sigma` is satisfactory small. Optional repeat a final time with the original data if the signal to noise of the original data permits.

8. Select only the images for stitching where the average of the used weights (i.e. where $w > w_{min}$) is larger than q_{thresh} for an appropriate value of q_{thresh}.

9. (Optional) For those images, match the intensities by calculating the intensity ratios between the overlap areas of size `fftsize*fftsize` and perform a global optimization.

10. Define a weighting mask, 1 in the center and sloping linearly to zero at the edges of the valid region, over a width of `bandwidth` pixels, as illustrated in Figure 15.

11. Per block of output `blocksize*blocksize`, select all images that have overlap with the particular output block, multiply each by the weighting mask and shift each image appropriately. Divide by an equivalently shifted stack of weighting masks. As such information at the center of images gets prioritized, and transitions get smoothed.

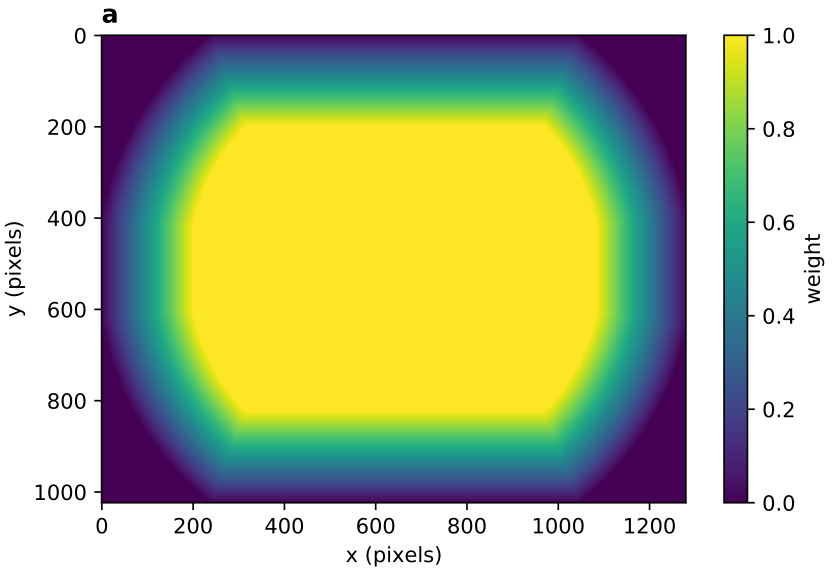

Supplementary Figure 15: Weight mask used to stitch images. *A linear slope of the weight towards the edges of the round microchannel plate detector is used to smoothly merge images.*

Considerations

For square grids with a decent amount of overlap, it makes sense to put `n_neighbors` to 5 (including the image itself), however, for larger overlaps or datasets where an extra dimension is available (such as landing energy), it can be appropriate to increase the number of nearest neighbors to which each image is matched.

Parameters and intermediate results of the iteration are saved in an `xarray` and saved to disk for reproducibility.

Parallelization

Using `dask`, the following steps are parallelized:

- step 5B , where each pair of images can be treated independently. In practice parallelization is performed over blocks of subsequent images with their nearest neighbours. This could be improved upon in two ways: firstly by treating each pair only once, and secondly by making a nicer selection of blocks of images located close to each other in the nearest neighbor graph. This would most likely require another (smarter) data structure than the nearest neighbour indexing matrix used now.
- Step 6 is quite analogous to 5B and is parallelized similarly.
- Step 11 is parallelized on a per block basis. To optimize memory usage, results are directly streamed to a `zarr` array on disk.
- The minimizations are parallelized by `scipy` natively.

Supplementary references

1. Kemao, Q. Two-dimensional windowed Fourier transform for fringe pattern analysis: Principles, applications and implementations. *Optics and Lasers in Engineering* **45,** 304–317. doi:`10.1016/j.optlaseng.2005.10.012` (2007).
2. Benschop, T. *et al.* Measuring local moiré lattice heterogeneity of twisted bilayer graphene. *Physical Review Research* **3.** doi:`10.1103/physrevresearch.3.013153` (2021).

3. Kerelsky, A. *et al.* Maximized electron interactions at the magic angle in twisted bilayer graphene. *Nature* **572,** 95–100. doi:10.1038/s41586-019-1431-9 (2019).
4. De Jong, T. A. & van der Molen, S. J. *pyGPA* Zenodo, 2021. doi:10.5281/zenodo.5589555.
5. Lam, S. K., Pitrou, A. & Seibert, S. *Numba* in *Proceedings of the Second Workshop on the LLVM Compiler Infrastructure in HPC - LLVM '15* (ACM Press, 2015). doi:10.1145/2833157.2833162.
6. De Jong, T. A. *TAdeJong/LEEM-analysis: LEEM-analysis* version 0.2.0. Zenodo, 2021. doi:10.5281/zenodo.5223927.
