# Supplementary material for: Imaging moiré deformation and dynamics in twisted bilayer graphene
Source: Nat Commun. 2022 Jan 10;13:70. doi: 10.1038/s41467-021-27646-1 (PMC8748992; doi:10.1038/s41467-021-27646-1)
Supplement: Supplementary file 3 — Description of Additional Supplementary Files [file 41467_2021_27646_MOESM3_ESM.docx]

**Description of Additional Supplementary Files**

**Supplementary Video 1:** Full movie showing larger Field of View compared to Figure 4 of the main text, in real space LEEM data, difference data and GPA-extracted displacement field.
